# Supplementary material for: Risk Factors for Recanalization after Coil Embolization
Source: J Pers Med. 2021 Aug 14;11(8):793. doi: 10.3390/jpm11080793 (PMC8398571; doi:10.3390/jpm11080793)
Supplement: Supplementary file 1 [file jpm-11-00793-s001.zip › Supplementary material 28.04.2021 Computational fluid dynamics analysis.pdf]

Risk factors for recanalization after coil embolization.

Karol Wiśniewski[1], Zbigniew Tyfa[2], Bartłomiej Tomasik[3,4], Piotr Reorowicz[2], Krzysztof Jóźwik[2], Ernest J. Bobeff[1], Bartłomiej J. Posmyk[1], Ludomir Stefańczyk[5], Marlena Hupało[1], Dariusz J. Jaskólski[1].

[1]Department of Neurosurgery and Neurooncology, Medical University of Lodz, Barlicki University Hospital, Kopcińskiego 22, 90-153 Lodz, Poland

[2] Institute of Turbomachinery, Lodz University of Technology, Medical Apparatus Division, Wolczanska 219/223, 90-924 Lodz, Poland.

[3]Department of Biostatistics and Translational Medicine, Medical University of Lodz, 15 Mazowiecka St., 92-215 Lodz, Poland

[4]Department of Radiation Oncology, Dana-Farber Cancer Institute, Harvard Medical School, Boston, MA 02215, USA

[5]Department of Radiology-Diagnostic Imaging, Medical University of Lodz, Kopcińskiego 22, 90-153 Lodz, Poland.

Corresponding author

Karol Wiśniewski,

Department of Neurosurgery and Neurooncology, Medical University of Lodz

Kopcińskiego 22, 90-153 Lodz, Poland

+48(042)6776770

karol.lek@poczta.fm

Supplementary material

6. Computational fluid dynamics analysis

6.1 Model geometries preparation

To identify a recanalization marker and to evaluate the influence of the aneurysm and its morphological parameters on the flow hemodynamics within the investigated domain, several geometries were created with the use of SolidWorks software. Each model was based on a referential (parent) case which was parametrized. It means that this parent geometry was prepared in such a way that the authors could change the desired morphological parameter, i.e. parent vessel diameter (*P*), aneurysm neck width (*N*), aneurysm height (*H*) and aneurysm max.

width (*S*). Afterwards, the program automatically updated the geometry basing on new parameters. Therefore, the spatial alignment and overall shape of the parent vessel, aneurysm and its bifurcation angle were maintained in each analyzed case. The only parameters that changed were those of utmost importance, i.e. morphological parameters described earlier in this study. Due to such an approach, the authors could analyze changes in flow hemodynamics resulting only from the differences in desired parameters.

Concerning the parent geometry preparation, the authors used an algorithm called 'extrusion by profiles', both for the parent artery and the aneurysm. However, profiles used for the artery reconstruction were circular, whereas for the aneurysm they were approximated for the patient-specific case. The aneurysm neck cross-section was modelled as an ellipse, where a larger diameter was equal to the user's input for the parameter *N*, whereas a smaller diameter was equal to 80% of this value. To preserve a proper spherical or oval shape of the aneurysm, its geometry was extruded by a few separate profiles. The second profile was distant from the first one (aneurysm neck) by a value equal to one-fifth of the user's input for the parameter *H* (0.2**H*), while the second profile, corresponding to the largest aneurysm cross-section, was distant from the previous one by 40% of *H* (0.4**H*). The consecutive profile was distant from the former one by 0.2**H*, whereas the next profile was distant by 95% of 0.2**H*. Hence, the remaining part of the aneurysm (5% of 0.2**H*) could be created as a properly-rounded and smoothed ending.

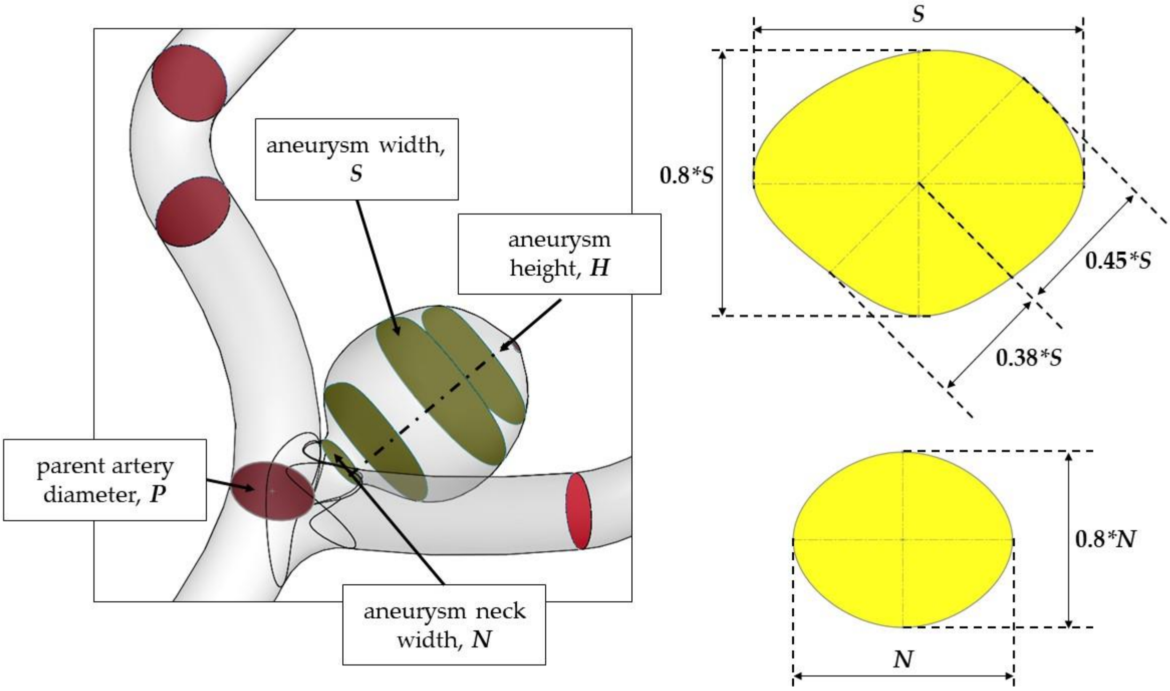

Supplementary Figure S1. Outlines an example model of the parametrized reconstruction together with images of the aneurysm neck and aneurysm largest cross section.

6.2 Numerical simulations

Flow solutions were obtained within Ansys CFX package by solving Reynolds-Averaged Navier-Stokes equations (RANS), which decompose the instantaneous field variables into time-averaged and fluctuating parts (Equations (S1) and (S2)). In general, Navier-Stokes equations describe the motion of fluids by applying the conservation of mass, momentum and energy theorems.

$$\rho \cdot \frac{\partial U_i}{\partial t} + \rho \cdot \frac{\partial}{\partial x_j}\left(U_i \cdot U_j\right) = -\frac{\partial P}{\partial x_i} + \frac{\partial}{\partial x_j}\left(2 \cdot \mu \cdot S_{ij} - \rho \cdot \overline{u_i' \cdot u_j'}\right) \qquad \text{(S1)}$$

where U_i U_j is mean part of velocity, $u_i' u_j'$ is the fluctuating part of velocity, P is the pressure field, while S_{ij} is the mean strain-rate tensor, expressed by:

$$S_{ij} = \frac{1}{2} \cdot \left(\frac{\partial U_i}{\partial x_j} + \frac{\partial U_j}{\partial x_i}\right) \qquad \text{(S2)}$$

In order to solve the three unknown quantities obtained after the aforementioned decomposition, a turbulence model had to be added. For the blood flow simulations, the golden standard is claimed to be k-ω Shear Stress Transport (SST) turbulence model. It provides sufficient turbulence approximations both in the boundary layer and in the free flow regime.

Since the authors assumed no heat exchange, the simulated flow was set as adiabatic and isothermal one. The blood was modelled as a non-Newtonian, incompressible fluid characterized by a power law viscosity model, governed by following equations:

$$\begin{cases} \mu = 0.554712 \;\; for \;\; \dfrac{\partial \mathrm{v}}{\partial \mathrm{y}} < 1e^{-9} \\ \mu = \mu_0 \left(\dfrac{\partial \mathrm{v}}{\partial \mathrm{y}}\right)^{n-1} \;\; for \;\; 1e^{-9} \le \dfrac{\partial \mathrm{v}}{\partial \mathrm{y}} < 327 \\ \mu = 0.00345 \;\; for \;\; \dfrac{\partial \mathrm{v}}{\partial \mathrm{y}} \ge 327 \end{cases}$$

where: μ – dynamic viscosity; μ_0 – reference dynamic viscosity (equal to 0.035 Pa·s), $\frac{\partial v}{\partial y}$ – shear strain rate; n – constant (equal to 0.6).

Before conducting target numerical simulations, a mesh independence test was carried out to ensure the most optimal meshing parameters. The main objective of this test was to assess whether the mesh is of sufficient quality and that any further mesh densening (i.e. generating meshes consisting of more and more elements) does not influence the obtained results. The

final meshes in each analyzed case consisted of circa 4.5 million elements.

The final numerical analyses were performed as transient (with pulsatile boundary conditions) and stationary simulations, where the latter served as initial conditions for the former ones. As far as transient analyses are concerned, they enabled simulating four full cardiac cycles with a time step equal to 0.01 s. Such a number of pulses ensured negligible variation of hemodynamic parameters between the latter heart cycles and independence from the initial boundary conditions. Graphical representation of the time-dependent boundary condition imposed on the inlet cross-section is depicted in Figure 2.

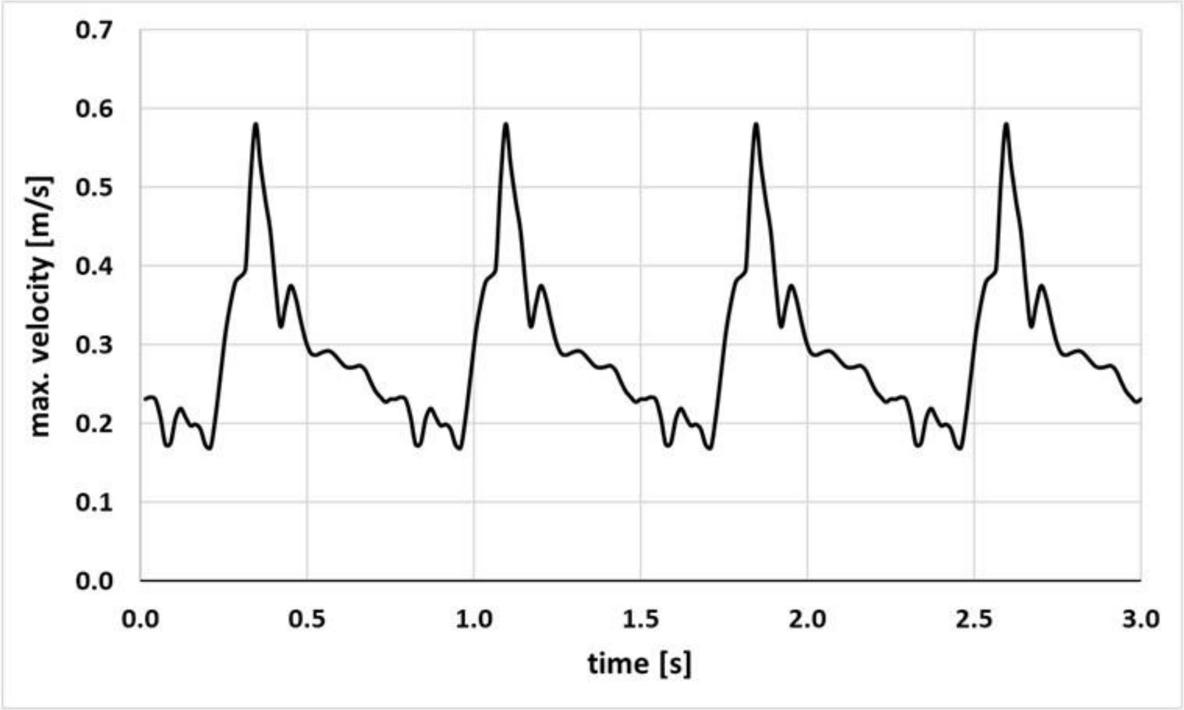

Supplementary Figure S2. Depicts time-varying velocity boundary condition used in each unsteady simulation.

The results analysis was focused on an examination of numerous hemodynamic parameters, including: time-averaged wall shear stress (TAWSS), vorticity, flow structure, oscillatory shear index (OSI), and WSS (wall shear stress).

9. *Computational fluid dynamics analysis*

Supplementary Table S1. Morphometric parameters used for reference case studies

	P [mm]	N [mm]	H [mm]	S [mm]
**Unruptured Reference**	**4.6**	**4.0**	**13.0**	**9.3**
**Ruptured Reference**	**3.52**	**3.6**	**12.0**	**12.0**

Supplementary Table S2. Morphometric parameters used during geometries reconstruction for CFD models

	Values which increase recanalization risk, based on ROC curves analysis	Case	High risk of recanalization				Low risk of recanalization			
			P [mm]	N [mm]	H [mm]	S [mm]	P [mm]	N [mm]	H [mm]	S [mm]
**Unruptured group**	S > 9.3 mm	**#1**	4	3	10	10	4	3	10	8
	H > 13 mm	**#2**	4	3	14	14	4	3	12	10
	N > 4 mm	**#3**	4	5	10	10	4	3	10	10
	P > 4.6 mm	**#4**	5	3	10	10	4	3	10	10
	SR ratio (H/N ratio) > 2.759	**#5**	4	3	30	10	4	3	20	10
	N/P ratio > 1.042	**#6**	3	4	10	10	4	3	10	10
	aspect ratio (H/N ratio) > 10.526	**#7**	4	3	28	10	4	3	20	10
**Ruptured group**	H > 12 mm	**#1**	4	3	20	10	4	3	8	10
	N > 3.6 mm	**#2**	4	4	10	10	4	2.5	10	10
	aspect ratio (H/N ratio) > 3.075	**#3**	4	3	10	10	4	3	8	10
	N/P ratio > 1.023	**#4**	3	4	10	10	4	3	10	10
